# Representation of Jews and Anti-Jewish Bias in 19th Century French Public Discourse: Distant and Close Reading

**DOI:** 10.3389/fdata.2021.723043

**Published:** 2022-01-26

**Authors:** Simon Levis Sullam, Giorgia Minello, Rocco Tripodi, Massimo Warglien

**Affiliations:** ^1^ Department of Humanities, Ca’ Foscari University of Venice, Venice, Italy; ^2^ Department of Management, Ca’ Foscari University of Venice, Venice, Italy; ^3^ Strategic Management Department, IESE Business School, Barcelona, Spain; ^4^ Department of Modern Languages, Literatures, and Cultures, University of Bologna, Bologna, Italy

**Keywords:** antisemitism, word embedding, jews, distant reading, natural language processing, bias, race, France

## Abstract

We explore through the lens of distant reading the evolution of discourse on Jews in France during the XIX century. We analyze a large textual corpus including heterogeneous sources—literary works, periodicals, songs, essays, historical narratives—to trace how Jews are associated to different semantic domains, and how such associations shift over time. Our analysis deals with three key aspects of such changes: the overall transformation of embedding spaces, the trajectories of word associations, and the comparative projection of different religious groups over different, historically relevant semantic dimensions or streams of discourse. This allows to show changes in the association between words and semantic domains (referring e.g. to economic and moral behaviors), the evolution of stereotypes, and the dynamics of bias over a long time span characterized by major historical transformations. We suggest that the analysis of large textual corpora can be fruitfully used in a dialogue with more traditional close reading approaches—by pointing to opportunities of in-depth analyses that mobilize more qualitative approaches and a detailed inspection of the sources that distant reading inevitably tends to aggregate. We offer a short example of such a dialogue between different approaches in our discussion of the Second Empire transformations, where we mobilize the historian’s tools to start disentangling the complex interactions between changes in French society, the nature of sources, and representations of Jews. While our example is limited in scope, we foresee large potential payoffs in the cooperative interaction between distant and close reading.

## 1 Introduction

The analysis of long-term changes in culture, and how they affect patterns of conflict and integration in society, has found new opportunities with the advent of large digital collections of textual data (Garg et al., 2018; Kozlowski et al., 2019).

In this paper, we address a largely debated issue in the history of religious, cultural, and political conflict: the rise of the “Jewish question” in modern France after 1789 and the birth of modern antisemitism during the long 19th century^1^ up to the First world war. France has been considered as the cradle of the antisemitic ideology that has tragically shaped the history of Europe in the 20th century, from Hannah Arendt’s *The Origins of Totalitarianism* (1951), to George L. Mosse, *Toward the Final Solution*. *A History of European Racism* (1980). The 19th century has also been the time in which communication media such as newspapers have become a crucial arena of political debate and conflict: a period in which the public sphere was structured and began to function in modern ways (Calhoun, 1992) anticipating elements of the so-called information society (as sociologist Manuel Castells has called it). Indeed, the Dreyfus case is one of the first examples of how the strategic use of printed communication media could polarize society, shape important changes in society and institutions, with concurrent phenomena such as the rise of an engaged public opinion of committed intellectuals (Charle, 2020).

**FIGURE 1:**
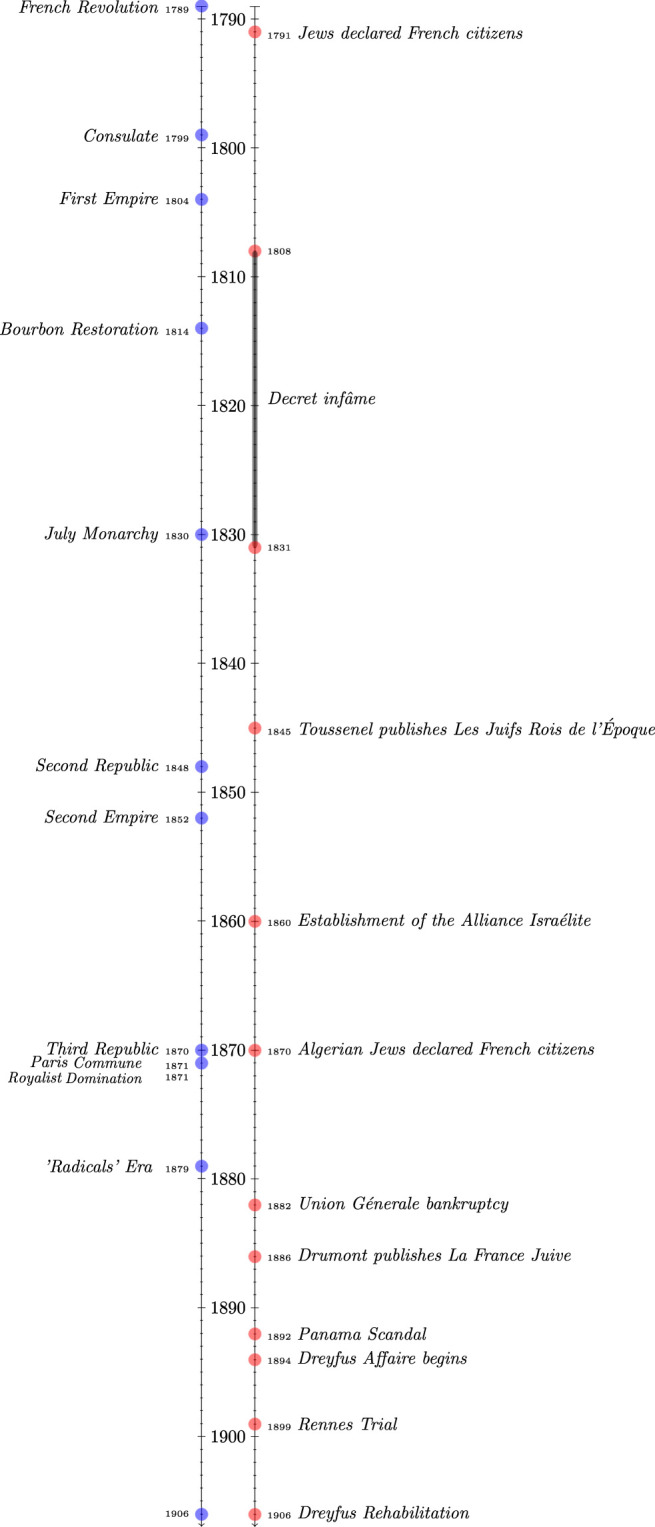
A timeline of the French long 19th century. On the right-hand side main jew-related events.

In this paper, we explore the evolution of discourse on Jews in France through a large corpus of printed French textual materials, 1789–1914, made available by the major digitization efforts of the Bibliothèque Nationale de France and its Gallica initiative^2^. Our analysis has been conducted by constructing diachronic word embedding spaces and tracing changes in the meanings associated to Jews in such spaces. A previous analysis based on the ARTFL Project (American Research Treasury of French Literature) database had limited itself to note the relevant frequency of references to Jews (*Juif, Juifs, Juive, Juives*) in the pre-revolutionary and revolutionary literature, without an actual further characterization of such references (Schechter, 2003). Analyses over such a long period of time are rich of methodological challenges—how to make embedding spaces comparable over time, how to trace the evolution of “meaning”, how to detect discontinuities in discourse, or how to manage the heterogeneity of sources. A further challenge is faced in this article: i.e. how to integrate the distant reading perspective enabled by textual big data analytic tools with the close reading approaches which have belonged to the historians’ approach for some time (Moretti, 2013; Lansdall-Welfare et al., 2017).

In this paper, the integration of distant and close reading goes in two directions. On one hand, the historian’s knowledge and interpretations provide the context and the some of the initial questions that inform our analytical efforts. On the other hand, we try to show that distant reading generates indicators and suggestions for further opportunities for qualitative historical research approaches by investigating in depth specific historical periods often under-explored or previously not consider.

## 2 Analyzing Narratives About Jews in French Periodicals and Books

### 2.1 The Corpus

The analyses performed in this paper are based on a corpus collected selecting documents from Gallica, the digital library of the Bibliothèque Nationale de France (BNF). Gallica is the largest digital library of digitized French materials including books, booklets, catalogues, periodicals. Digitized books amount overall to over 690, 000 items, periodicals reach almost 4 million items.

The selection includes periodicals and books that contain a keyword related to Jews (Supplementary Material for the complete list of keywords) and have been published between 1789 and 1914. We have identified through historiography words strongly characterized as expressions of traditional religious or economic antisemitic streams (below Section 4).

The period 1789–1914 corresponds to the beginning of the entry of the Jews into French society (1791) and precedes the First world war and especially the publication in most European languages of the infamous forgery the “Protocols of the Elders of Zion”, which represented a major turning point in the history of antisemitism.

The research was further restricted to those resources that have an OCR quality higher than 98*%* as reported by the BNF digitization process. Even if previous work Hill and Hengchen (2019); van Strien et al. (2020) have demonstrated that lower OCR thresholds do not affect the corpus analysis results, we decided to keep our corpus as clean as possible, avoiding to create representations of misspelled words. The resulting corpus contains 54, 403 books and 245, 188 periodicals issues (we included in the corpus also documents that contain just a single keyword). To control the representativeness of the corpus we checked if the main newspapers of the time are covered by our selection. We discovered that more than 80% of all the issues of a selected list of newspapers (Supplementary Material Section 4) can be discovered in our corpus, including popular newspapers such as “Le Petit Parisien” (775, 000 readers) and less popular newspapers such as “La France” or “Le Pais” (1, 000 readers).

**FIGURE 2:**
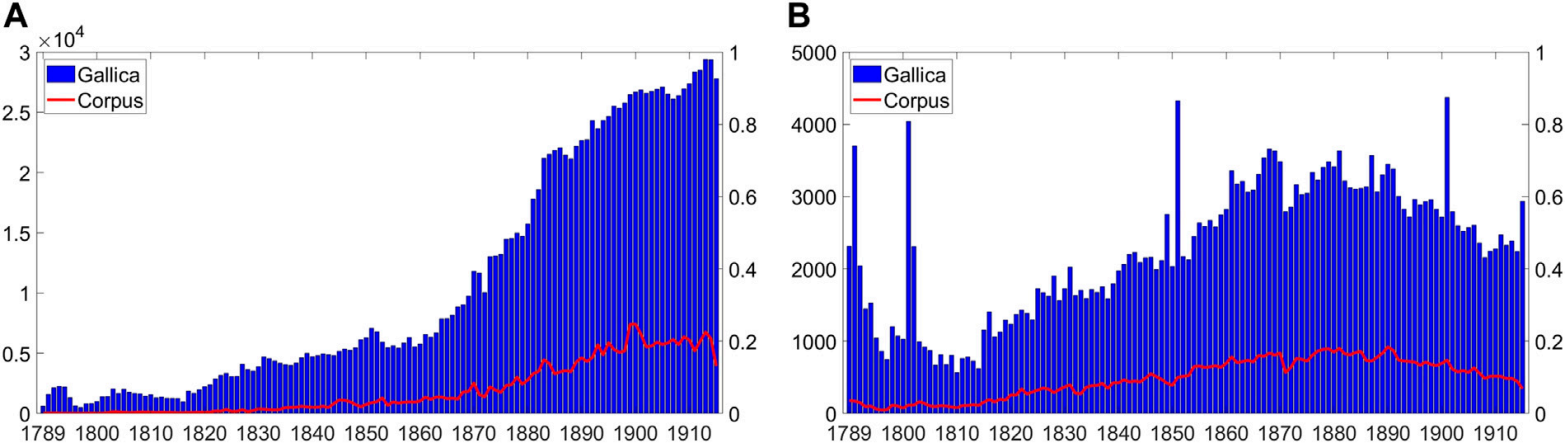
Distribution of resources per year in the **(A)** periodicals and **(B)** books sub-corpora. The charts present values on both y-axis sides: on the left numbers indicate actual quantity whereas on the right fraction of corpus data with respect to the whole Gallica data (red line).

Figures 2A,B indicate the distribution of resources per year in the periodicals’ and books’ sub-corpora respectively, together with the total number of resources in Gallica as of June 2018 (*y*-axis values on the right-side refer to fraction of our corpus with respect to the entire Gallica). The resources distribution per year is not homogeneous in neither sub-corpora: publications increase significantly year by year. Several explanations can be put forward about the growth of documents over time. First, there is the fact that the print industry grew gradually in the period considered. In fact, many publishers and newspapers were founded after 1825: Hachette, for example, the publisher with the largest number of books in our corpus (1, 558), was founded in 1826. The national daily *Le Figaro*, was founded in 1826, the Catholic *L’Univers* in 1833, while the liberal *La Presse* was established in 1855, same year as the conservative daily *Journal des débats politiques et litéraires*, and *Le Temps* was created in 1861. Another key element probably relates to the growing importance of Jews in the French public discourse, later culminating in the proliferation of anti-Semitic newspapers such as the ultra-Catholic *La croix*, Edouard Drumont’s *La libre parole*, *La lutte antijuive* and *L’intransigeant*, to name a few. At the same time it is important to notice that the Jewish population of France remained relatively stable in its numbers in the course of the Nineteenth-century, counting approximately 40,000 people, i.e. 1*%* of the 40 million total French population.

Figures 2A,B, plotting our corpus compared to the whole Gallica corpus for the period considered, seem to suggest that the first factor (the growing publishing industry) was the most relevant: in fact the quantity of resources in our Jewish-related corpus follow a trend similar to those observed in the whole Gallica corpus from the period we considered.

### 2.2 Word Embeddings for Historians

Analyzing large diachronic corpora to study historical changes in the meaning of words requires choices on how to formally represent meaning. Word embeddings have proven that a spatial representation of lexical items can offer productive insights into the meaning of words and concerning how such meaning changes over time (Garg et al., 2018; Kozlowski et al., 2018). The key representational assumption is that the meaning of worlds is captured by their co-occurrence relations—the meaning of a word is defined by the context in which it is used (Harris, 1954; Weaver, 1955; Firth, 1957). The second assumption is that unique words can be represented as points having coordinates in a high-dimensional geometric space. To respect the first assumption, words that share many contexts should be close to each other in such a space.

Word Embedding is an umbrella term for techniques converting textual data into such spatial representations. More precisely, words of a natural language are being transformed into high-dimensional vectors whose proximity reflects similarity in the contexts they are associated with.

There are two main approaches to construct word embeddings: 1) frequency-based embedding (Pennington et al., 2014) and 2) prediction-based vectors (Mikolov et al., 2013a,b). While the former is quite intuitive, as it relies on the count of words in documents, the latter, the one adopted in our analysis, needs more clarification. As explained above, the basic idea is that linguistic items with similar meanings tend to be used in similar contexts. Thus, the aim is to derive a vector representation of lexical items to preserve semantic relations by constraining words with similar contexts to have similar representations.

These methods form an *n*-dimensional representation of a word through an artificial neural network with one hidden layer between the input and the output layer. In layman’s terms, a neural network is a set of computation units laid out in one or more layers, connected with each other, in order to mimic some features of brain behavior. Notably, there are weights responsible for transforming input data within the network’s hidden layers. These weights are the learnable parameters of the network.

Word2Vec (Mikolov et al., 2013b) comes in two flavors. If the network input is a group of words, i.e. the context, the technique is the CBOW (Continuous Bag of Words) whereas if we feed the network a single word the method is called Skip-Gram. Skip-gram methods learn the embedding by predicting the surrounding words given a current word, whereas CBOW predicts the target word given a context.

In this work we built word embeddings using the skip-gram approach. The basis of word2vec *via* Skip-Gram is a shallow network that has as output a prediction vector, with a probability of each word in the vocabulary to occur near the input word.

It should be noted that the training samples for the model are generated by sliding a window of n-word over the text (that is for each word the *n*-words that come before the input word and the *n*-words that come after it). As the model is focused on learning about words given their local neighbors, the context is provided by such a window of nearby words. The size of the window is a crucial parameter of the model as it affects the vector similarities. For instance small windows are inclined to highlight syntactic similarities while larger windows capture topical similarities (Goldberg, 2017).

Though the skip-gram model trains a neural network to perform a prediction task, the network outputs are not used. Instead, the network is trained to learn the weights of the hidden layer, which are actually the “word vectors”.

There are diverse benefits to using word embedding methods. For instance, they take less memory to save and are more computationally efficient, compared with other vector representations, such as one-hot encoding: while one-hot vectors are high-dimensional and sparse, since they have the size of the vocabulary, word embeddings are lower-dimensional and dense (Bengio et al., 2003). This characteristic in turn allows larger embeddings to be learned. Besides, it has proven that these word vectors have the ability to capture similarity between words, for instance by using cosine similarity.

Diachronic word embeddings (Kulkarni et al., 2015; Hamilton W. L. et al., 2016; Kutuzov et al., 2018) are constructed by training embeddings on homogeneous corpora over different (but sequentially concatenated) time spans, producing embeddings for each time period independently. Given the stochastic nature of word embedding models it is necessary to align such embedding spaces in order to make them comparable. The standard approach to align word embeddings consists in using the orthogonal Procrustes alignment. This approach, given two matrices, in our case the learned low-dimensional word embeddings, finds an orthogonal matrix that most closely maps the first matrix to the second one. In this way, it is possible to make comparisons across word embedding, e.g. compare the use of the word *juif* in two different time periods.

Diachronic word embeddings have been mainly used to compute two distinct measures on word embeddings, i.e. global measure of change and local neighborhood measure of change (Hamilton WL. et al., 2016). The former can be used to analyze time series of pairwise similarity between words, to control specific shifts in meaning over time (an approach that we use in Section 3); the latter is used to measure changes in a single time period. However, an interesting question concerns overall shifts in the space structure over time—that may help to detect overall transitions in meaning systems. We explore this issue, that has received less attention until now, and propose a measure that can capture overall variations in embedding spaces to characterize semantic divergences interpreted as isometric deviations.

Given the extreme time dispersion of the first time slot, in most analyses we use only time slots after 1830. For each time period we trained a word2vec skip-gram model Mikolov et al. (2013b) using a window size of 5 words on both sides, a word vector of 300 dimensions and removing the words that occur less than 25 times. To limit the effects of training embedding models on different quantities of data we decided to divide our data in time bins of approximately the same size in terms of tokens (∼ 500 million).

## 3 Measuring Large-Scale Changes in Meaning Systems

To address the problem of large-scale historical changes in the geometry of meanings we introduce a new tool for diachronic embeddings evaluation, derived from topological data analysis (TDA) (Wasserman, 2018). This technique has been demonstrated to be effective in bilingual lexical induction to measure to what extent two embedding spaces in different languages can be aligned (Patra et al., 2019). Again, some technical notions need to be introduced. TDA is a set of tools that can detect structure and shape in data in order to obtain valuable information from the data domain. Data often encode information yet even more often this information is very challenging to extract (e.g. due to complexity or noise). In order to understand geometric structures of data, it is suitable using TDA and its methods, such as persistent homology (Edelsbrunner and Harer, 2008)− actually a branch of topological data analysis. In the NLP field, Zhu (2013) was among the first to introduce an algebraic method based on Persistent Homology, specifically to provide a new document structure representation^3^.

Following Patra et al. (2019), Michel et al. (2017) and Zhu (2013), we adopt Gromov-Hausdorff distance and use an approach based on persistent homology to assess how well two embedding spaces can be aligned under an isometric transformation. Basically, we aim at comparing embeddings based on their intrinsic geometric properties.

The Hausdorff distance between two metric spaces is a measure of the “worst case” or the diametric distance between the spaces (Rockafellar and Wets, 2009). It measures the distance between the nearest neighbours that are the farthest apart. Formally, Hausdorff distance from set X to set Y is a *maximin* function defined as

$$H\left(X,Y\right)=\underset{x\in X}{\max}\;\left\{\;\;\underset{y\in Y}{\min}\;\;\left\{\;d\left(x,y\right)\;\right\}\;\right\}$$ (1)

where *X* and *Y* are two non-empty subsets of a metric space and *d*(⋅,⋅) is a distance function. More precisely, the complete definition is

$$H\left(X,Y\right)=\max\left\{\underset{x\in X}{\sup}d\left(x,Y\right),\underset{y\in Y}{\sup}d\left(X,y\right)\right\}$$ (2)

In Eq. (2) *sup* is the *supremum* (i.e. the least upper bound of a set), and *d* (*x*, *Y*) quantifies the distance from a point *x* ∈ *X* to the subset *Y*.

So, given two sets of points, the Hausdorff distance is computed as follows: 1) calculate the distance between *x*_*i*_ and *y*_*j*_s; 2) among these distances keep the shortest one; 3) repeat steps 1) and 2) for all *x*_*i*_; 4) among the shortest distances find the largest one. In practice, the Hausdorff distance is the maximum distance of a set to the nearest point in the other set (Rote, 1991).

Unfortunately such a distance cannot be directly utilized for our goal as it is not isometries invariant (namely in case of isometries, such as translations, reflections or rotations, the distance is not preserved). Nevertheless, there exists a more suitable distance, the Gromov-Hausdorff (GH) distance Mémoli (2008). The GH distance minimizes the Hausdorff distance (Eq. (1)) over all isometric transforms, providing a quantitative isometry of the two spaces (an isometry is a transformation that preserves distances between metric spaces). In other words, the GH distance, applied to word embeddings, is the minimum Hausdorff distance under all possible isometric embeddings.

$$GH\left(X,Y\right)=\underset{f,g}{\inf}\;\;H\left(f\left(X\right),g\left(Y\right)\right)$$ (3)

where *f* and *g* belong to the set of isometric transforms and *inf* is the *infimum*, that is the greatest lower bound in the set. Intuitively, the GH distance measures how far two sets are from being isometric. We say that two embeddings have the same geometry if *GH* (*X*, *Y*) = 0, i.e. if they are the same up to an isometry. Consequently, the greater is the distance the more dissimilar are the two embeddings.

Computing the GH distance is still intractable for large embedding spaces, despite many efforts in this area (Bronstein et al., 2006). Following Chazal et al. (2009), we approximated it by computing the bottleneck distance between the two metric spaces, a concept based on persistent homology. The bottleneck distance measures the similarity between two persistence diagrams, as it is the shortest distance in order that there exists a perfect matching between points of the two diagrams. A persistence diagram is a scatter plot of 2-D points showing the appearance (birth) and disappearance (death) of certain topological features, such as holes, empty hulls or connected components, under varying resolutions (it is like replacing each point by a sphere of increasing radius).

In our experiments, for each time slice, we created word2vec word embeddings and then measured the distance between two spaces by approximating the GH distance, that is we measured how far two metric spaces are far from being isometric or, equivalently, preserve distance between elements. More precisely, we measured the distance between vector spaces induced by the neighborhoods of the words *juif* and *juifs*. Basically, instead of using the whole vocabulary, which is the entire word embedding, namely the full set of word vectors, we considered only those 500 terms close to the words *juif*, *juifs*, by cosine similarity. These terms, in turn, define a neighborhood, a subset of all word vectors, or equivalently a vector space induced by the neighborhoods of the words *juif* and *juifs*.

**FIGURE 3:**
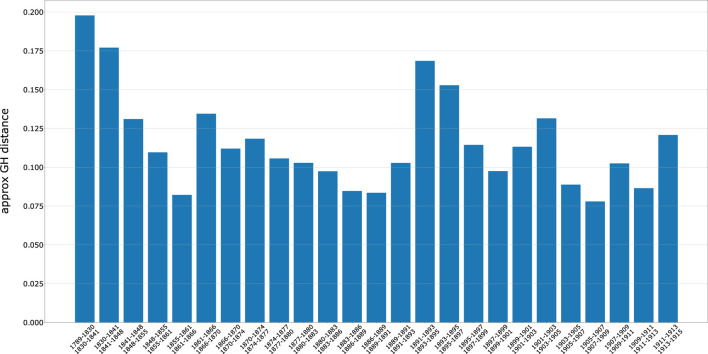
Distance between word embeddings of consecutive time slices calculated using the approximated Gromov-Hausdorff distance. The greater the distance the more dissimilar the two embeddings. The distance captures the amplitude of changes in the geometry of the embeddings, from period to period. Concurrently, it provides an overall measure of semantic change happening over time.

Figure 3 shows, in bar plot format, the approximated GH distances between word embeddings of sequential time slices. In essence, each bin is the distance between the two vector spaces belonging to the periods indicated in the *x*-axis. Such a distance captures the amplitude of change in the geometry of the embedding, from period to period. It offers an overall measure of semantic change happening over time.

For the historian, this approach points to periods in which relevant changes are unfolding. Unsurprisingly, a peak of changes happens in the period in which the synthesis of modern political antisemitism, inaugurated by Edouard Drumont and others, starting in the mid 1880s, translates into a visible social movement, accompanied by the emergence of an openly and aggressive antisemitic public discourse (Angenot, 1989). These years include the editorial and press campaigns targeting Jews in the second decade of the Third Republic, the antisemitic outburst provoked the Panama scandal in 1892 (which had actually been preceded by the 1882 *krach* of the Catholic bank Union Générale, followed in turns by an antisemitic crisis (Verdès-Leroux, 1969), and the subsequent deflagration of the Dreyfus affair (1896). However, two relevant periods of change which have been hitherto less investigated by historians who have worked on the image, representation and role of Jews in French Nineteenth-century society are highlighted by our analysis. The first one coincides with the decline of the Restoration, the preparation and explosion of the revolution of 1848 and the emergence of the Second Republic. The Second period is that of the Second Empire and especially the 1860s, which end with the Paris Commune followed by the establishment of the Third Republic.

In Section 6 we use such an indicator of change to begin an investigation of the evolution of the representation and discourse on Jews during the Second Empire, that shows how distant reading can fruitfully lead to and actually require a shift to close reading of digitized historical documents and of their specific contexts.

## 4 The Dimensions and Evolution of a Bias: Between Stereotype and Antisemitic Offence

The trajectories of words in diachronic embedding spaces can be used to explore the evolution of word meaning over time (Hamilton W. L. et al., 2016). Changing distances between words correspond to shifts in semantic associations. Here we look at changes in distance between two focal words - *juif* and *juifs*, henceforth *juif(s)*—and a set of words that have characterized the stereotypical characterization of Jews in France especially in the course of the 19th century (Supplementary Material). In this way, we try to capture how discourse on Jews, its bias (or biases) and its antisemitic components evolved in the course of over a century.

We organize our presentation categorizing words by four streams which have often been recognized by historians as constitutive of antisemitic thought in the 19th century: the religious, the economic, the conspiratorial, and the racial streams of antisemitism (Wilson, 1982). We suggest however to describe them, more generally as streams of bias (refining what we did previously in Tripodi et al. (2019), as they are not necessarily the result of straightforward antisemitic expressions, but can be associated to discourse going from stereotype (including literary representations considered only today stereotypical or even antisemitic) to prejudice, to outspoken antisemitic offences (including accusations of conspiracy). To clarify this distinction we should first add historical perspective to our analysis. In fact, we cannot classify or measure antisemitism on a historical basis exclusively from our post-Holocaust perspective, since what is mostly a linguistic, cultural, political and religious tabu in the discourse on Jews today, was not considered as such at the time. To be more explicit: the kind of discourse we are dealing with also clearly did not have the same bloody consequences as Nazi or Fascist anti-Jewish discourse and propaganda from the 1930s and 1940s (Herf, 2008).

We should also keep in mind, given the relevance of literary texts in our corpus, that stereotypical representations of Jews, including negative and positive representations, also when connected in different ways to the history of the centuries-old anti-Jewish prejudice, were part also of the Western literary tradition, including literature of the highest standards from Shakespeare to Céline (and literature produced and spread by Jews or partly Jewish authors) [most recently Samuels (2018)].

The religious dimension is of course a defining aspect of the representation of Jews in 19th century French public discourse as in previous and following periods and not only in France. Unsurprisingly the nearest neighborhood of both *juif* (s) in the embedding space is populated by words directly referring to Jewish religion and religious institutions (e.g.: *Jahvè*, *rabbin* etc.). Moreover, other religions populate largely such space. However, religion is also associated with the stereotyping of Jews as religious opponents and enemies of Catholicism and to negative connotations of the Jewish of faith. Religious antisemitism, typically of Catholic origin, is the major stream of antisemitic discourse since the middle ages and contributes to defining its shape and nature also in the modern period (Nirenberg, 2013).

**FIGURE 4:**
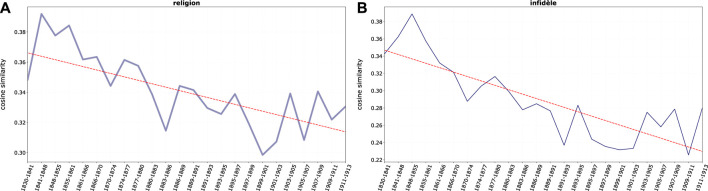
Average cosine similarity between **(A)** *juif*, *juifs* and religious terms and **(B)** *juif*, *juifs* and single term *infidèle*.

Frequent examples of negative words associated to Jews are, in our corpus: *impie* (impious), *mécréant* (miscreant), *infidèle* (infidel), *pécheur* (sinner). Such terms display high cosine similarity with the focal words *juif* and *juifs* particularly at the beginning of the 19th century. Remarkably, our data show a declining trend (with some fluctuations, as shown in Figure 4) of such elements in time, despite the clear effort and tendency to revive religious factors in the modern “antisemitic synthesis” attempted by Drumont and his allies (Kauffmann, 2008; Judaken, 2011). Still while religious antisemitic language remains important, it seems to lose importance at least in explicit public discourse (i.e. in printed material) in an increasingly secularized society.

A different pattern emerges when looking at another important stream of discourse typically associated with Jews: the economic discourse. Since the middle ages, Jews have been associated consistently to a stereotype of trade and moneylending, with associations to *argent* (money) and *usurier* (usurer) as the dominant ones, but also to *marchand* (merchant) and to dishonest commercial practices (*gueusard* being the most prominent example, very close to *juif(s)* at the beginning of the century). *Prostitution* has often been associated as well to Jewish women as part of a world of small businesses at the borders of morality.

**FIGURE 5:**
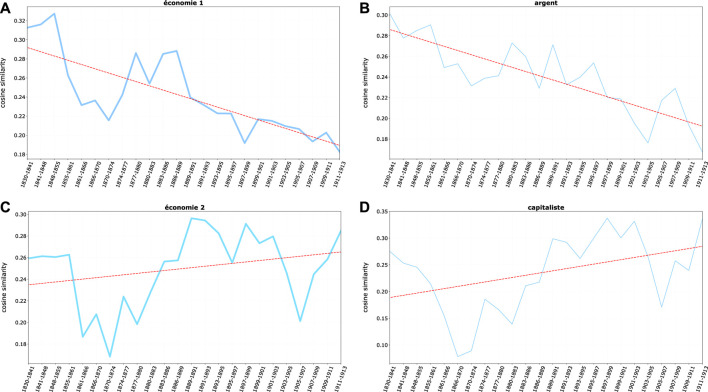
Average cosine similarity between **(A)** *juif*, *juifs* and first group of economic terms, **(B)** *juif*, *juifs* and single term *argent*, **(C)** *juif*, *juifs* and second group of economic terms, and **(D)** *juif*, *juifs* and single term *capitaliste*.

**FIGURE 6:**
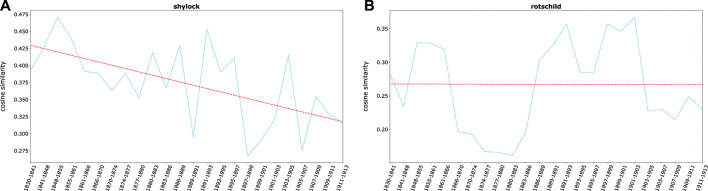
Average cosine similarity between **(A)** *juif*, *juifs* and single term *shylock* and **(B)** *juif*, *juifs* and single term *Rotschild*.

All these terms display a clear downward trend (Figures 5A,B), although usury-related words remain constantly among the nearest neighbors of *juif(s)*—the medieval stereotype still preserving its strength despite a relative decline. The literary expression of such archaic economic stereotype, *Shylock*, as shown in Figure 6A, follows a similar fate. While being always strongly associated to *juif (s)*, it slowly fades in time - although one should notice that representation and even the public success of Shakespeare’s play is not in itself a sign of the spread of antisemitic beliefs nor is it necessarily motivated by anti-Jewish feelings (on *Shylock* see, for example, Shapiro (2016)).

In parallel, a remarkable shift in the economic semantic domain happens, mirroring the emergence of a Jewish economic (mostly financial) *élite*, well integrated in the French bourgeoisie and connected to political powers. *Banquier* (banker) and *capitaliste* (capitalist) are the words better capturing the emergence of this new economic *élite* (Figure 5B). Both words went across two major cycles of relevance gains—a cycle shared by *Rotschild*, symbol of Jewish economic and social prominence, Figure 6B (on the Rothschild in France a major source of the period is Dairnvaell (1846); it has been analyzed by Kalman (2010); among the historical literature Bouvier (1992); Ferguson (2000); Marcilhacy (2016). On the “Rothschild effect”, Savy (2010)).

The first period—which we consider in detail below in Section 6—coincides with the French Second Empire (1851–1870), when the spectacular ascent of the Pereire brothers, but also of the Fould brothers (prominent both in the economy and in politics), seem to eclipse the ascent of the Rothschild family, until their sudden fall in the last years of Napoleon III government. These were also the years of the construction of the French railways infrastructure, of the founding of the stock exchange market, of the transformation of the urban landscape of Paris through the creation of its boulevards, and also of a growing cultural, social and economic integration of Jews into the French society (Cohen, 1980; Nord, 1995).

The second cycle coincides with the years of the “opportunist radicals” political cycle, and moves into the Belle Époque, traversing the Panama scandal (1891–1892) and the key Dreyfus years (1894–1900). Both cycles go hand in hand with the rise of a new attribute of the Jewish stereotype: “*exploiteur*”, a clear token of the re-emergence of a economic and socialist economic aversion to Jews, which had seen a first ascent in the post-revolutionary years (Battini, 2016) but also in the 1830s and 1840s peaking in the publication of the later classic Alphonse Toussenel’s *Les Juifs rois de l’époque* (1845 and 1847, reissued in 1886 and 1888) (Crapez, 2014).

Thus the economic discourse on Jews in France seems to undergo a complex transformation in which, during two major cycles, the old stereotypes lose weight (while remaining relevant) and a new stereotype, carrying both the signs of successful integration and of new hostilities, slowly takes its place, with strong cyclical fluctuations.

Another important shift in the discourse on Jews takes place during the 19th century, leading gradually to the future major crisis of the *Troisieme République*, the Dreyfus affair (1894–1906). It is the rise of a conspiratorial view of the Jews. Together with the association to the financial *élite*, this stream of discourse will also be a major ingredient of the first forgery of major and tragic historical consequence, the “Protocols of the Elders of Zion”: a prominent bloody anticipation of fake news, which has been called a “warrant for genocide” (Cohn, 1996).

**FIGURE 7:**
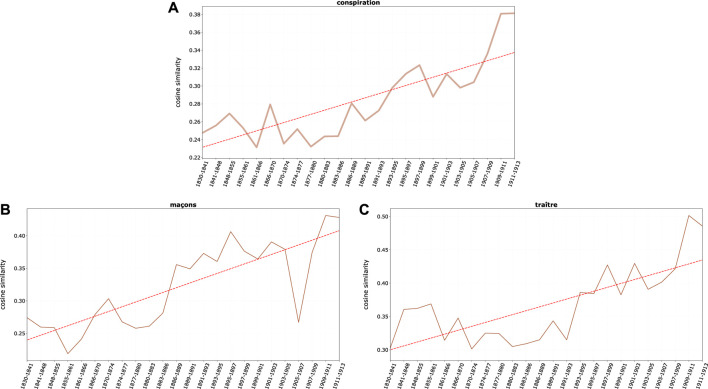
Average cosine similarity between **(A)** *juif*, *juifs* and conspirational terms, **(B)** and **(C)** *juif*, *juifs* and single term *maçon* and *traître*, respectively.

While some of the conspiratorial themes (*complot, conspiration*) seem to gain prominence mostly since the 1880s, also as a result of the campaign of agitators as Drumont and his followers–and so does the strong association of *juif(s)* and *franc-maçon* (thanks to the rise of the freemasonry, Katz and Oschry (1970), it is worth noting that the identification of the *juif* as a *traître* (traitor) becomes a growing tendency in the nationalistic spirit of the second Empire years, a period which, once again, still further deserves scrutiny (Figure 7). The theme of the *juif* as an *espion* (spy), that would play such an important role in the Dreyfus case, precedes the antisemitic movements, as it is spurred by the Franco-German war of 1870 (Angenot, 1994)). Still, one cannot forget the theme of the eucharist desecration and, especially, of the blood libel from the preceding centuries (Rubin, 2004; Teter, 2020).

**FIGURE 8:**
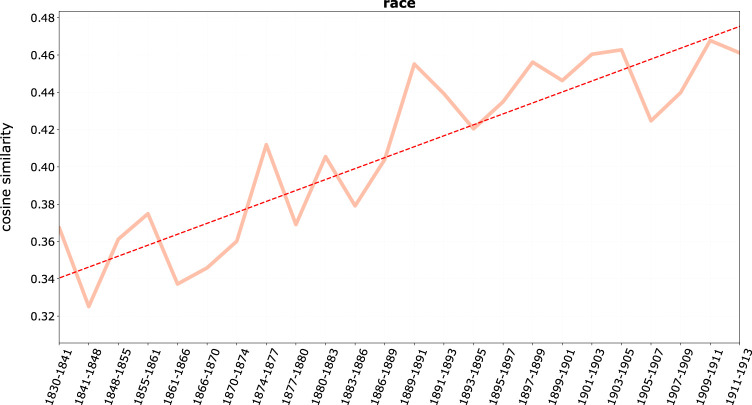
Average cosine similarity between *juif*, *juifs* and race terms.

The fourth stream of bias we have identified is race, shown in Figure 8, displaying a steady trend of increasing proximity to *juif(s)* in the course of the century.

This suggests a slow substitution of “race” for religion as identifying factors in discourse on French Jews (Leff, 2005). This is also consistent with a secularization process of French society, with the growth of racial thinking in historiography and in the rising social sciences in the last decades of Nineteenth-century.

## 5 Biases: A Comparison of Four Religions

The geometric structure of word embeddings also provides an intuitive way to analyze the existence of biases hidden in the use of language. The basic idea is that it is possible to construct within the embedding space axes representing a continuum of qualities between extreme polarities in specific semantic domains, such as for example masculine-feminine or poor-rich. These axes can be constructed very intuitively as differences between the vectors representing the semantically polar words.

For example, if “pious” and “impious” are represented in a given embedding space by the vectors w_pious_ and w_impious_, w_I_ = w_impious_-w_pious_ will capture an “impiety” axis in the embedding space.

To make those axes semantically more robust, one may construct them as the average difference between different pairs of polar words (i.e. antonyms). For example, one may add unfaithful-faithful, disbeliever-believer and so forth and create an average axis capturing degrees of religious quality. It is then possible to “locate” different words on such axes by a projection operation—the projection can be computed as the dot product w_i_⋅w_p_ of a word *i* vector with the semantic domain axis vector. The intuition is that the higher the projection value, the closer the world will be to the left extreme of the semantic axis. For example, by constructing an axis representative of the male-female domain, one can project different sports words on such axis, and obtain (as expected) that in English language boxing is closer to the masculine pole than softball (Kozlowski et al., 2018). Projections have been used to detect gender or racial bias (Bolukbasi et al., 2016; Caliskan et al., 2017) in word embeddings. Kozlowski et al. (2018) have suggested that they can be used in a diachronic analysis to capture the evolution over time of cultural meanings.

In a similar vein, here we explore the use of projections to compare bias across the major religions of 19th century France (Catholics, Protestants, Muslims and Jews). We compare them across multiple semantic domains that capture important aspects of discourse on Jews during such period (Wilson, 1982). A few of them have been already analyzed in the former section, to delineate the evolving stereotype of Jews through key words in four semantic domains: the religious, economic, racial and conspiratorial ones. In all these dimensions, pairs of antonyms capture the negative/positive valence of attitudes and behaviors associated to different religions. So, for example, the economic dimension captures the evaluation of economic behavior in terms of unfairness/fairness and greed/altruism. To those we add the morality and political loyalty sphere. The online supplement reports the pairs of antonyms used to construct the different dimensions. All these discourse streams are captured by sets of pairs of antonyms that qualify opposite qualities in the relevant domain (Supplementary Material). The antonym pairs have been selected starting from a word that is highly representative for the stream, subsequently using a knowledge base to collect its synonyms and the corresponding antonyms—this reduces potential selection bias effects.

We project both the singular and plural name of religious groups (e.g. Jew and Jews). We keep separate the analysis of singular and plural to reflect a concern for the phenomenon of *singularization* (Miccoli, 2003)—i.e. the characterization of *the* Jew as an enemy through the use of the singular to make generalizing and often stereotypical statements about Jews. A cautionary word is in order before we proceed with our analysis. Our comparison is based on a corpus of French texts that have been selected because they contain words associated to Jews. Thus, the corpus represents a subset of the whole set of available French publications in the reference period. What we measure, thus, is bias relative to the discourse on Jews—which anyway is our object of analysis.

**FIGURE 9:**
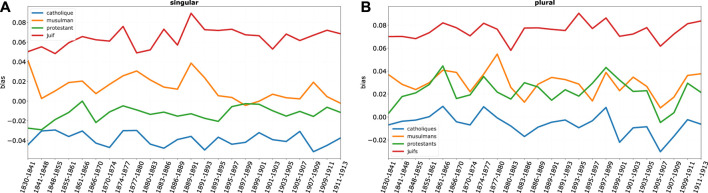
Overall bias, four religious groups, based on projections (Supplementary Material). Higher values represent larger negative bias.

Before looking at biases in single streams, it is useful to compare the overall position of different religious groups on a negative-positive continuum, as shown in Figure 9. If we sum for each group the biases on each stream, we obtain a clear ranking of religious groups. Jews are by far those most affected by a negative bias; furthermore, negative bias against Jews shows a progressive increase over time, until the eight-hundred nineties, especially for *the Juif*. However, levels of overall bias stabilize in subsequent years. Moreover, while relative bias - the relative difference between religions—is more accentuated for the singular, absolute bias - the absolute bias value—is higher for *juifs* than for *juif* until ca. 1870—then differences tend to disappear. Thus, *singularization* seems to be a more complex process than usually supposed. The growth of negative bias precedes to a large extent the explosion of antisemitic movements in the last decade of the century. On the other hand, Catholics clearly stand on the positive side of the bias spectrum. Quite interestingly, however, Muslims are second by negativity but not too distant from protestants, and actually levels of overall negative bias are very close between the two groups when the plural term (Muslims, Protestants) is considered—again, bias is more accentuated under singularization.

**FIGURE 10:**
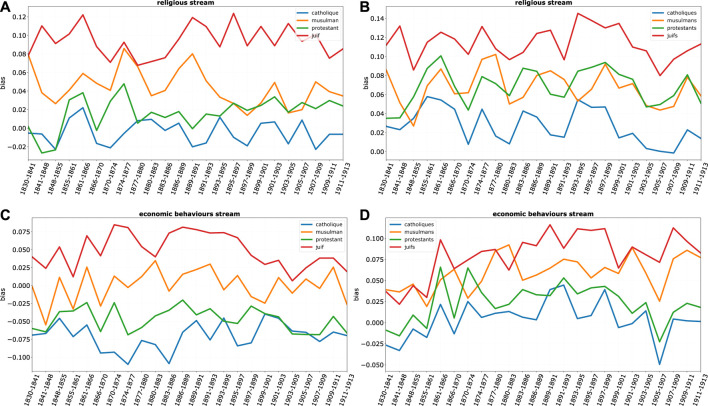
Bias, for religious groups, by stream, where each row is a stream: religious and economic (**A–D)**. On the **(A,C)** bias for the singular term (e.g. *Protestant*) while on the **(B,D)** for the plural term (e.g., *Protestants*).

**FIGURE 11:**
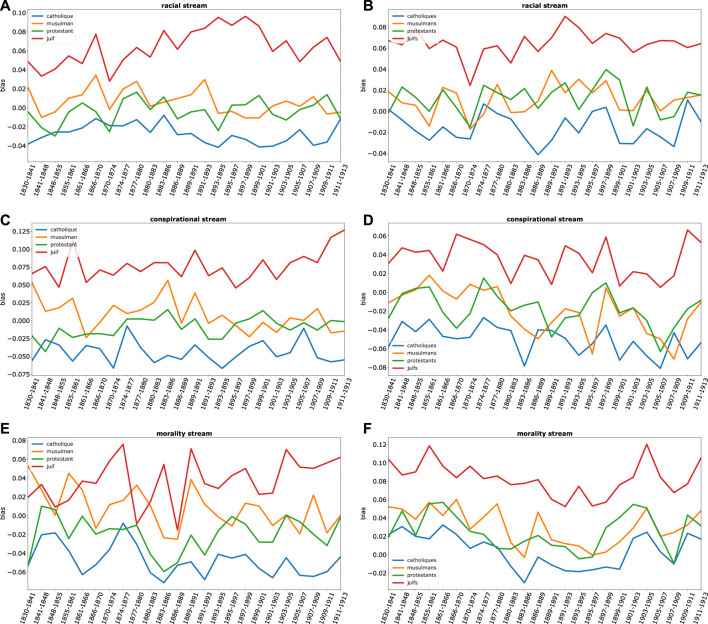
Bias, for religious groups, by stream, where each row is a stream: **(A–F)** racial, conspiratorial and morality. On the **(A,C,E)** bias for the singular term (e.g. *Protestant*) while on the **(B,D,F)** for the plural term (e.g., *Protestants*).

This result can be better understood if we look at the decomposition of the overall bias by discourse streams (Figures 10, 11: again, higher values correspond to more negative bias). While on all streams Jews are those most affected by negative bias, and Catholics have a mirroring positive position, bias on Protestants is heavily differentiated by domains. While protestants enjoy an almost neutral position in most streams of discourse, they suffer a remarkably negative bias in the religious domain. This seems to show that despite the great social ascent of protestants, the long shadow of protestant minorities religious prosecution is still present in 19th century France.

Other domains reveal some interesting phenomena. A noteworthy example is again the economic domain. Figures 10C,D show important differences in the economic stream between singular and plural terms. Economic bias on Jews is much amplified when the plural term is considered. In this case, the Jew is seen less unfavorably than Jews as a collectivity are the subject of discourse. The ascent of economic “plural” bias is especially remarkable in the period preceding the Dreyfus case. However, this is a phenomenon affecting not only Jews but also the other religious minorities, which might suggest a broader cultural shift in attitudes towards the economic behavior of different social groups. The most remarkable difference concerns once more Protestants. While “the” Protestant is definitely on the positive side of economic discourse all along the 19th century, Protestants as a collective do suffer consistently over time of a negative bias, especially in the first part of the century, aligning them with the other minorities.

Finally, two additional comments on the racial and conspiratorial streams (Figures 10G,I). Projections suggest an important fact: that Jews are the only religious group consistently associated with negative racial connotations—while other groups lie substantially on a neutral line or are definitely associated to positive racial bias in the case of Catholics. This suggests a unique characterization of the race-religion connection in antisemitic terms.

As far as the conspiratorial stream is concerned—on which we already focused attention in the former section of this paper—it is worth noticing that it is again uniquely associated to Jews, but with a strong differentiation between the singular and plural terms. In sharp contrast to the economic domain, here bias is mostly associated with *Juif*. While negative economic forces are more impersonal and collective, the conspirator is singularized.

## 6 From Distant Reading to Close Reading: A Look at the Second Empire

### 6.1 Historical Context

The period 1850s–1860s, coinciding with the French Second Empire, has often been neglected by the historiography of French and modern antisemitism more generally with very few exceptions (for the context Hazareesingh (2014); on the Jewish question in this period, Isser (1991); Kertzer (2008); a different attention has been given to the 1840s, Frankel (1997).

A big data perspective through text analysis and historical embeddings has to the contrary pointed our attention to the relevance in this period of the representation of Jews and of references to Jews, both positive and negative, though with a prevalence of the latter in the literature (all sorts of printed materials) and in the press of the time.

The material which is more prominent in our corpus associated with Jews is to begin with literary, consisting of about 30 authors of mostly work of fiction. These went from Alexandre Dumas and Victor Hugo to the very popular genre of *feuilleton*, often including authors today largely forgotten except by literary experts and cultural historians of the period. The relevance of this literary material must be carefully scrutinized and interpreted as it cannot be considered plainly and at first sight as representing an “antisemitic moment” (to quote and paraphrase Birnbaum 2011 and Birnbaum 2015) even when it displays the association of negative terms of various sort and contains stereotypical representation of Jews, both negative and positive. One should note, incidentally, however that also positive and philosemitic representations of Jews can be stereotypical and not necessarily friendly, for example the image of a supposed Jewish superior intelligence (Gilman, 1996).

The 1850s corpus displays—as we already noticed - mostly two semantic streams of bias associated with “the Jew” or “Jews”: religious language and economic language (often again with conspiracy theory undertones). The first is unsurprising since the linguistic, semantic and symbolic discursive materials available for any anti-Jewish critique or attack in the first half of the 19th century were mostly religious and deriving in various forms from the centuries-old tradition of Christian and especially (particular in the case of France) Catholic antisemitism Katz (1980); for a detailed reconstruction of the relations between State and Church in France for the period we are examining Debidour (1898), more recently Ford, 2005; on the Second Empire Maurain, 1930.

The second—economic—stream associated with the Jews and mostly stereotypical and hostile is also not a complete innovation, since the accusation of usury, greedy attitudes etc, is also profoundly engraved in the representation of Jews and in anti-Jewish prejudice and hatred (including theological anti-Judaism). This was originated by the forced function of money lenders attributed to Jews in the middle ages, the early modern period and the Ancien Regime more generally, deriving also from the Canon Laws and the religious doctrine of the Catholic Church prohibiting Christian to perform any activity of money lending with interest. One should also consider the legend of the Jewish role in the invention and spread of the “bill of exchange”, analyzed by Trivellato, 2019.

The novelty of the use of economic language associated with Jews, which actually emerged already in counter-revolutionary and reactionary thinkers like De Bonald in the aftermath of the French Revolution (Battini, 2016), is connected to the emerging transformations especially of the trends in French capitalism since the 1840s and 1850s. We already mentioned: the emergence of the railway system and the entrepreneurial role in that context of some capitalist family of Jewish origins as especially the Rothschild; the emergence and definition of the new stock exchange market also with a Jewish role; the presence and function, largely exaggerated and emphasized in the political discourse, of Jewish entrepreneurs and also of Jewish politicians at least since 1848 (starting from Adolphe Cremieux). On the economic and financial side a relevant and visible presence was that of the Pereire and of the Fould family and especially of Achille Fould who became also Napoleon III’s minister of Finance for some time (Cousteix, 1950; Landes, 1956).

This period and the role of the Rotschild in particular started in the 1840s in economic and political pamphlets and in literature, with a prominent role of Balzac and his *La Comedie Humaine* (1830–1856) (Méchoulna, 2005) and of the so-called “Rothschild effect”: this was the phantasmic and ghostlike exaggeration of the role of Jewish capitalism in the France, England, the West, with various figures and branches of Rothschild bankers and entrepreneurs.

The critique of the Jewish presence in the economy developing and changing since the 1830s and blossoming in the 1840s and 1850s, derives from a socialist tradition remounting especially toFourier who was in competition with the Saint-Simonian movement (characterized by a relevant Jewish presence). This grew and became first crystallized in the mid 1840s with the publication of the later very influential Adolphe Toussenel’s *Les Juifs Rois de l’Epoque* (resurrected and made famous in the 1880s by Toussenel’s personal friend and follower Edouard Drumont), which intertwined fourierist socialism and reactionary Catholicism. Toussenel had in fact also been close to Eugene Vieullot, a central figure in reactionary French Catholicism (and a former Protestant converted to Catholicism), in particular as editor of the influential Catholic ultra-conservative and ultra-montane newspaper *L’Univers*.

It should be noted that Veuillot became in the late 1850s one of the protagonists of the international scandal of the Mortara affair. Edgardo Mortara was a 9-year-old Jewish boy from Bologna kidnapped by the Pontifical Army, brought to Rome and forcibly converted inside the walls of the Vatican where he would later become and remain for the rest of his life a Catholic priest. This episode created an international uproar partly comparable to the much larger resonance of the Dreyfus Affair 40 years later. These affairs were preceded in the international public opinion by the so-called Damascus Affair of 1840 (Frankel, 1997), a famous blood libel accusation of European wide and especially French and Italian resonance, involving the disappearance in Syria of a Catholic priest supposedly massacred by Jews for the use of his blood in Easter or Passover rituals - according to the centuries old antiJudaic and conspirationist accusation.

These important episodes, which certainly had a profound resonance in the imaginary and language of the time, Catholic and not, represented the impact of the processes of secularization and the decline of the traditional faith, but also a reaction to the advancement of modernity, anti-clericalism and rationalism. At the same time there was in this period, especially starting in the 1850s, a return of the religious with new cults of Saints and pilgrimages (the blossoming of Lourdes, the nationalist cult of Joan of Arc, and the proclamation of the dogma of the Immaculate heart).

### 6.2 The Sub-corpus and the Embeddings

The analyses in this section are based on a portion of our corpus described in Section 2.1 which includes only documents published in the time frame 1851–1871. This portion comprises 26, 709 periodicals and 14, 465 books in total and amount to roughly 9.5 gigabytes of compressed UTF-8 encoded textual files. The total number of documents digitized in Gallica for the same time frame amounts to roughly 230, 000. With these textual materials we created word embeddings using the word2vec skip Gram model (Mikolov et al., 2013b). We used a window size of 5 tokens, removed all the tokens that occur less than 5 times, ran 5 iterations and set the dimensions of the vectors to 300. With these parameters and data we were able to create embeddings for 398, 719 words.

Reading the list of books’ authors we noticed that the authors with the highest number of books in the corpus are mostly if not all, novelists. The top ranking authors in terms of number of books include Alexandre Dumas (114 books), Victor Hugo (58 books) and Eugène Sue (32 books), among others. Given the high number of books of fiction in the corpus we decided to also create a reduced version of the corpus, discarding all the books whose authors are in the top 30 authors ordered in terms of number of books. In such way we removed from the corpus roughly 1 gigabyte of data. The length of the vocabulary of this new corpus is 360, 241 and all the words in this vocabulary are also in the vocabulary of the entire embeddings.

### 6.3 Neighborhood Similarity

The first analysis we conducted is an evaluation of the neighbourhood of the words *juif* and *juifs* in the two sub-corpora. To compute the similarity among word vectors we used the cosine similarity. Despite its simplicity, this approach is stable and effective. In fact, as demonstrated by Gonen et al. (2020), analyzing the neighborhood of a word vector allows to identify words that are used differently in two corpora.

What we noticed from this analysis is that the first 50 words most similar to *juif* are related mainly to the religious domain, with words such as *talmudiste*, *synagogue*, *rabbin* and *prophète*: these are both descriptive, uncharacterized, but also derogatory terms (e.g. *talmudiste* and—under certain circumstances—*rabbin*, used for example as a synonym for *usurer*). Other words in the neighbourhood refer to proper nouns of personalities who were prominent on the French public scene of the period (Jewish or believed to be or represented as Jewish). This will happen frequently especially later when for example Drumont and others will consider or define as Jewish the French prime minister of Italian descent Léon Gambetta).

Interestingly, even when we removed from our corpus the most represented novelists, two words that characterize the neighbourhood of *juif* are *errant* and *laquedem*. Such terms are actually an adjective and a name, referring to the historical novel written by Alexandre Dumas by the title *Isaac Laquedem or Le Roman du juif errant* 1852)—so literature returns through references to it in other books and periodicals, confirming its role in the representation and textual presence of Jews. Dumas’ *Le Juif errant* is however a complex (and unfinished) serialized novel which both perpetuates certain anti-Jewish Catholic prejudices (the Jews as Christ-killers) and proposes a new heroic reinterpretation of the wandering Jew (Lyon-Caen, 2012). In general French literature of the time contains and spreads both prejudices and stereotypes and new forms of idealization and aestheticization (often stereotypical) about Jews, including Jewish females. This was the case, for example, of an important literary and artistic success of the mid-decades of the century often emerging in our data: the popular opera *La Juive* by Jacques Halévy, which debuted in 1835, and was subsequently very frequently performed in the 1840s and 1850s (and actually up to the 1930s). Its female protagonist was a typical and stereotypical *belle juive* (beautiful Jewess), but also the symbol of a woman persecuted on religious grounds (Hallman, 2007; Samuels, 2009; Savy, 2010). While distant reading has allowed us to confirm the role of literature in the emergence and spread of the representation of Jews in this period and, we assume, of the spread of bias connected to the word *Juif*, we should underline that only close(r) reading can allow us to identify and investigate the specific sources of such representation and to study such texts and their contexts. These may reveal ambivalent representation of Jews: negative and even partly positive biases. A further step would require a study of the reception of such texts by contemporaries and permit to establish—for example from reviews in newspapers of the time or other contemporary testimonies (diaries, letters etc.)—whether, how and to what extent the biases contained in the texts were treated, accepted or refuted. Finally, this closer reading should allow us to formulate reasonable hypotheses on the relationships between texts and contexts: i.e. to further investigate to what extent contexts generated those texts and contributed to their success; whether those texts represented popular attitudes and at the same time contributed to produce them, together with their related biases (Samuels, 2018).

#### 6.3.1 Streams Similarity

Another measure that we computed through word embeddings is the average cosine similarity among the words *juif* and *juifs* and a list of words that define our six streams of bias. This list was created by selecting from the embeddings the 500 words most similar to *juif* and *juifs*. Each word in this list was then manually associated to one of our 6 streams^4^.

We noticed that this measure is consistently higher for the word *juifs* than for the word *juif* in all the streams (Table 1) and we suggested that the plural noun gathers more bias through the embeddings, as the plural generalizes “Jewish” attitudes which are in such way referred to a collectivity [It should be recalled however that also the use of the singular *Juif* has been considered a generalizing or “singularizing” term (Miccoli, 2003)].

**TABLE 1:** Average cosine similarity among the words *juif* and *juifs* and the words defining the six streams of bias; sub-corpus with and without literature.

*Stream*	*All*		*No Lit*	
	Juif	Juifs	Juif	Juifs
Religious	0.37	0.52	0.37	0.52
Economic	0.25	0.31	0.24	0.30
Socio-political	0.25	0.25	0.25	0.24
Racist	0.24	0.25	0.23	0.25
Conspiratorial	0.26	0.28	0.21	0.24
Ethic	0.17	0.21	0.15	0.21
Attributes	0.36	0.23	0.35	0.22

Another interesting finding is that the only stream that has similar values for both the singular and the plural is what we have defined as the conspiratorial stream. The conspiratorial and the religious stream will come together towards the end of the period we are considering with the publication of a text usually considered of great relevance in French antisemitic literature of the second half of the Nineteenth-century Des Mousseaux 1869 (the book was republished in France in 2005). This text displays mostly a traditional ant-Judaic attitude, but it actually also emphasizes in new ways the conspiratorial dimension, while at the same time referring to historical events related to the Emancipation of the Jews of France and insisting especially in chapter on the recent antisemitic vague in Romania of the years 1864–1866 (see Isser pp. 117–122; on the book’s spread including by Nazi translators, see Frankel, p. 419; on its influence on the “Protocols of Zion” Taguieff, 2004).

If we analyze the cosine similarity of the words *juif* and *juifs* with words related to the economic domain we can see also that in this case the use of the plural has a higher similarity with all the terms, and that there are some terms that are more similar than others, e.g. *bourgeois*, *bourgeoisie*, banque, *marchand*, *monnaie*, *privilège*, *finance*, *prêt* and *argent*. This persistent connection established between references to Jews and terms which have to do with the economy, the market, banking, stock exchange is certainly due to the increasing presence and especially the visibility—but also the polemic and biased emphasization—of Jewish entrepreneurs in French society in the 1850s–1860s. There is also a growing presence in this period of literature expressing moral views about economic transformations (Fourcade and Healy, 2007; Berger and Przyrembel, 2019), at times also in connection with Jews (Karp, 2008); unfortunately an important study of French economic language of the time, including terms used in our investigation which we have analysed in relation to *Juifs*, does not include the noun *Juif* (Dubois, 1962).

### 6.4 Sentiment Analysis of the Neighborhood

We adopted a lexicon-based sentiment analysis perspective to better analyze the semantic space in which our target words, i.e. *juif* and *juifs*, are immersed. To this end, we first selected the 1, 000 most similar words to each target and then lemmatized them using the LEFFF lemmatizer (Sagot, 2010). The lemmata obtained can then be used to retrieve the period’s or the vocabulary’s sentiment from a predefined curated lexicon. For this purpose we used the French Expanded Emotion Lexicon (FELL) (Abdaoui et al., 2017).

It contains more than 14, 000 distinct words expressing emotions and sentiments. The words in it are labeled using the Ekman basic two polarities and six emotions. It has been created using a semi-automatic approach. First the English Emotional Lexicon NRC—Canada (Mohammad and Turney, 2013) has been automatically translated and expanded, then the data has been manually validated by professional translators. From this experiment we can see that for both target words the majority of similar terms have a negative connotation and that the corpus without literature has more terms (Table 2). From this experiment we can see that for both target words the majority of similar terms have a negative connotation and that the corpus without literature has more terms. The list of terms we obtained is available here on a dedicated repository:

**TABLE 2:** Number of positive/negative terms close to words *juif* and *juifs*; sub-corpus with and without literature.

—	*Entire Corpus*		*Corpus without Lit*	
	Negative	Positive	Negative	Positive
Juif	399 (55*%*)	319 (45*%*)	426 (52*%*)	382 (48*%*)
Juifs	501 (51*%*)	486 (49*%*)	552 (52*%*)	509 (48*%*)

https://github.com/roccotrip/long_century_bias.git

We suggest here only very preliminary findings, since this approach would require a much more detailed and refined investigation, looking once again at texts and contexts, and lending in itself to a another specific article. Analyzing the positive terms for both target words we noticed that such terms do not have a clear polarity and in most of the cases are not adjectives. The positive terms include among other words general, but also unexpected terms, including for example: *peuple*, *voyageur*, *philosophe*, *polonais*, etc. and many religious terms. In general we may say that these are positive terms associating Jews with the intellectual and literary sphere, and with aspects of the political arena which are specific of the period: for example the rise of the Polish national question and of the fight for the unification and liberation of Poland (which incidentally were also characterized by a public discourse intertwining religious, biblical and modern political especially nationalist elements).

In the findings produced by this approach negative words appear to display a more defined polarity and include words such as, to give only a few, relevant examples: *païen*, *renégat*, *imposteur*, *diable*, *désolerait*, *trafiquant*, *misérable*, *espion*, *prostituée*. Such terms clearly indicate religious counterposition, conflict and prejudice, but also all the elements of the ethical, religious, economic and political biases we identified in our corpus as associated with Jews, including the theme of treason and conspiracy. Perhaps there is a more relevant ethical dimension emerging through this approach, concerning real or—more often—imaginary Jewish activities, misbehaviors and morality.

## 7 Conclusion

In this paper we have explored through the lens of distant and close reading a longstanding historiographical issue, the evolution of discourse on Jews in France during the XIX century as a historical case for the study of documents relating to religious, cultural, economic, social conflicts and opinion dynamics. We have analyzed a large textual corpus including heterogeneous sources - literary works, periodicals, essays, historical narratives, political treatises, pamphlets - to trace how Jews are associated to different semantic domains, which we have called streams of bias, and how such associations shift over time. Our distant reading is conducted through a distributed, geometric representation of lexical items - diachronic word embeddings—that offers both an analytic (i.e. quantifiable) and a synthetic representation of semantic changes and shifts in the spatial location of words in the embedding spaces that we reconstructed.

Our analysis has dealt with three key aspects of such changes: the overall transformation of embedding spaces, the trajectories of word associations, and the comparative projection of different religious groups over different, historically relevant semantic dimensions or streams of discourse. This has allowed us to trace moments of semantic change, or more precisely to suggest the possible evolution of associations and stereotypes, and the apparent dynamics of anti-Jewish religious cultural and political bias over a long time span, during which dramatic institutional, political, economic and cultural changes unfold in France.

Our analysis confirms obvious elements of continuity and persistence over time, together with significant transformations in the composition of the multiple ways in which Jews are represented in printed textual sources in the period 1789–1914. On the one hand, our work confirms established historical reconstructions and interpretations of the history of the “Jewish question” in France and of French antisemitism, through a massive textual material that usually eludes qualitative methods of historical inquiry. On the other hand, our analysis points to hitherto under-investigated periods and aspects of time which have prepared in the course of the 19th century the well-known, visible and largely analyzed emergence of modern political antisemitism in the period 1880 ca to, and including, the Dreyfus affairs.

Distant reading through text analysis of big data has especially turned our attention to the period of the Second Empire (1851–1870). This period has generally been considered by historiography one of increasing integration of Jews into the political, social and economic fabric of French society. But our analysis has shown, confirming the hypothesis of a small number of historians who have looked at the period from this point of view, that this is actually a time when one can find the seeds of the rise of cultural and political conflicts around Jews that will characterize only 30 to 20 years later the Dreyfus affair—a major episode which has been considered a laboratory for the tragic developments of antisemitism in the 20th century.

However, a distant reading approach has also revealed some structural constraints in the analysis of such changes. We have therefore suggested that the investigation of large textual corpora should be fruitfully applied also in conjunction with more usual close reading approaches, through both digital and analogical i.e. qualitative methods. In-depth analyses can mobilize more qualitative approaches, i.e. a detailed inspection of the sources that distant reading inevitably tends to aggregate, but which is actually necessary to identify the “causes” and the details of textual concentrations. This also requires a careful scrutiny of the historical contexts—and not only of the texts—which produce the semantic streams (streams of bias) and the textual phenomena we observe.

The final section of this article, in particular, has offered a case study in the interaction between distant and close reading and an example of the necessary use of both approaches in the digital text analysis of large textual corpora. Close reading emerges to be also needed for a more complex interpretation and explanation not of the mere textual concentrations, trends of recurrences etc, but of the specific nature, contexts, genesis and evolution of streams of bias and of the concentration around particular topics. In our case the chosen topics are Jews in Nineteenth-century France and the history and development of anti-Jewish representations, and of biases and prejudices of various kinds and origins: religious, economic and, cultural.

A distant and close reading especially of the hitherto relatively unexplored or under-explored period of this history (the Second Empire), has offered us the possibility of synthetically describing the complex interactions between changes in French society, the nature of sources, and the representations of Jews, as we have mobilized both the historian’s tools and the new tools of digital text analysis and digital history. While our example is limited inscope, we foresee important potential results in the cooperative interaction between distant and close reading as digital and analogical methods are applied in the analysis of texts and contexts.

This has allowed us to describe and measure the developments of such phenomena, which emerge from the production of discourses and representations and from the interaction between texts of various nature and political, cultural, social and economic contexts and changes. Such phenomena are of course not limited to the Second Empire nor to Nineteenth-century France, but they can be observed more generally in the modern period, the public sphere, the so-called information society until today.

## Data Availability

The raw data supporting the conclusion of this article will be made available by the authors, without undue reservation.

## Footnotes

1 About the main events of the French 19th century please see timeline in Figure 1

2
https://gallica.bnf.fr

3 As the persistent homology is not the crux of this paper, we will not introduce a rigorous definition in this section. For more details we refer the reader to (Zhu, 2013)

4 The complete list of words and their classification can be found at https://github.com/roccotrip/long_century_bias
