# Supplementary material for: Representation of Jews and Anti-Jewish Bias in 19th Century French Public Discourse: Distant and Close Reading
Source: Front Big Data. 2022 Jan 26;4:723043. doi: 10.3389/fdata.2021.723043 (PMC8826090; doi:10.3389/fdata.2021.723043)
Supplement: Supplementary file 1 [file DataSheet1.pdf]

frontiers

Supplementary Material

1 KEYWORDS

List of keywords used to select periodicals and books and build the corpus. Please see Section 2.

- *Juif* (i.e. Jew - masculine, singular)
- Juive (i.e. Jew - feminine, singular)
- Judaisme (i.e. Judaism)
- Israëlite (i.e. Israelite)
- Israël (i.e. Israel)
- Israëlitisme (i.e. Israelitism)
- Mosaïsme (i.e. religions referred to the message of Moses)
- Talmud (i.e. Talmud)
- Judas (i.e. Judass)
- Moloch (i.e. the biblical name of a Canaanite god associated with child sacrifice)
- Ahasverus (i.e. a mythical immortal man whose legend began to spread in Europe in the 13th century. The original legend concerns a Jew who taunted Jesus on the way to the Crucifixion and was then cursed to walk the earth until the Second Coming.)

2 STEREOTYPE CHARACTERIZATION OF JEWS STREAMS

Words for the stereotypical characterization of Jews in France in the course of the Nineteenth century. Please see Section 4.

- Religious
  - ☐ *impie, mécréant, infidèle, pécheur*
- Economic 1
  - ☐ *argent, usurier, marchand, prostituée, gueusard, avare*
- Economic 2
  - ☐ *banquier, capitaliste, exploiteur*
- Conspiratorial
  - ☐ *conspiration, complot, maçons, maçon, traître, espion*
- Racial
  - ☐ *race, sémite*

3 BIAS PROJECTION AXES

Sets of pairs of antonyms qualifying opposite qualities, by discourse stream. Please see Section 5.

Religious

(spirituel, séculier), (sacré, profane), (pieux, païen), (pieux, idolâtre), (pieux, impie), (sacré, maudit), (vénérable, abject), (fidèle, infidèle), (croyant, incroyant), (religieux, irreligieux)

Economic behaviors

(donner, approprier), (générosité, cupidité), (générosité, avidité), (généreux, avide), (généreux, avare), (généreux, pingre), (prodigue, cupide)

Racial

(supériorité, infériorité), (estimable, laide), (affable, méchant), (estimable, infâme), (sympathie, haine), (accepté, refusé), (meilleur, pire), (pur, impur), (superieure, inférieure), (pur, infect), (propre, sale)

Conspiratorial

(loyal, espion), (honnêteté, trahison), (loyal, traître), (clair, mystérieux), (ouvert, occulte), (sincère, trompeur), (sincère, déloyal), (ouvert, secret), (amical, menaçant), (clair, obscur)

Morality

(décent, indécent), (vertueux, lascif), (moral, immoral), (honnête, malhonnête), (vertueux, corrompu), (chaste, dépravé), (chaste, charnel), (pur, dépravé), (honnête, canaille)

4 NEWSPAPERS

Table S1: List of newspaper outlets. The table contains also the number of readers of each newspaper and the number of issues in the corpus.

Title	#readers	#issues
L'Aurore	93.000	3352
La Lanterne	78.000	6659
Le Radical	70.000	4013
Le Rappel	30.700	4731
Le Siècle	20.000	4596
Le Signal	4.400	1321
Le Petit Parisien	775.000	4704
Le Figaro	54.000	4801
Le Temps	44.000.	4831
Le Matin	35.000	4816
Le Petit Journal	995.000	4731
La Croix	190.000	5548
Le Journal	180.700	4559
L'Intransigeant	141.000	4739
L'Écho de Paris	125.000	4691
La Presse	100.000	4712
Le Gaulois	23.000	4842
Le Journal des débats	16.800	5563
L'Univers	7.800	4918
Le Petit Caporal	6.900	4630
La Gazette de France	3.900	4626
La France	2.000	4406
Gazette nationale ou le Moniteur universel	1.400	2606
Le Pays	1.000	3673
